# A large-scale causal analysis of gut microbiota and endometriosis associated infertility: A Mendelian randomization study

**DOI:** 10.1097/MD.0000000000037383

**Published:** 2024-03-22

**Authors:** Yan Wang, Wangshu Li, Chunfang Ha

**Affiliations:** a General Hospital of Ningxia Medical University, Yinchuan, China; b Dalian Women and Children’s Medical Center (Group), Dalian, China

**Keywords:** causal inference, endometriosis, immunity, Mendelian randomization, sensitivity

## Abstract

Endometriosis is a prevalent condition with notable impacts on fertility. Recent studies have implicated gut microbiota in the development of endometriosis associated infertility (EAI). This study employs Mendelian randomization (MR) to elucidate the causal relationship between specific gut microbes and EAI. Using MR, we selected single nucleotide polymorphisms associated with 211 gut microbiota taxa from large-scale genome-wide association studies summary data. We applied statistical methods including inverse variance weighting, weighted median, and MR-Egger for analysis. Outliers were identified through the leave-one-out method. MR-Egger intercept tests were conducted to address horizontal pleiotropy, while Cochran Q and *P* values assessed heterogeneity. The false discovery rate method was used for multiple testing correction. Sensitivity analysis and *F* statistics evaluated the reliability and potential biases of our results. The inverse variance weighting method indicated a significant association of the genus *Actinomyces* (OR = 1.657, 95% CI: 1.187–2.312, *P* = .00298) with an increased risk of EAI. Conversely, genera Holdemania (OR = 0.630, 95% CI: 0.444–0.894, *P* = .00969) and Ruminococcaceae NK4A214 group (OR = 0.689, 95% CI: 0.481–0.999, *P* = .0439) appeared as protective factors. MR-PRESSO global test and MR-Egger regression indicated no significant horizontal pleiotropy (*P* > .05). Leave-one-out analysis confirmed the robustness of these findings. Our study provides evidence of a causal relationship between specific gut microbiome taxa and EAI. These findings offer novel insights and may guide the development of new preventive and therapeutic strategies for managing EAI.

## 1. Introduction

Endometriosis was previously considered a chronic gynecological disease. However, with further research, it is now defined as a complex systemic clinical syndrome, greatly impacting the reproductive health and quality of life of women of childbearing age.^[1,2]^ Studies estimate that the prevalence of endometriosis among women during their reproductive years is about 5% to 10%.^[3]^ Critically, endometriosis poses a significant challenge to the fertility of patients, with approximately 30% of those affected also experiencing infertility.^[3,4]^ Further investigations have revealed that up to 50% of infertile women are diagnosed with endometriosis.^[5]^ Compared to infertility triggered by other causes, women with endometriosis have a significantly reduced pregnancy rate (36% vs 55%).^[6]^ Additionally, it has been observed that women with endometriosis have poorer ovarian reserves, lower quality oocytes and embryos, and face challenges with embryo implantation.^[7,8]^

Although a large number of cases of endometriosis associated infertility (EAI) can be observed clinically, we still do not fully understand the pathogenesis of EAI. Currently, potential mechanisms for EAI are believed to include endometriosis subtypes, pain, inflammation, changes in pelvic anatomical structures, adhesions, ovarian reserve/functional disruptions, impaired endometrial receptivity, and the systemic effects of the disease.^[2,9,10]^ In recent years, research has identified connections between the gut or reproductive microbiota and various gynecological diseases, especially a link between gut microbiota and EAI.^[11,12]^ Dysregulated gut microbiota can trigger EAI by elevating pro-inflammatory cytokines, impairing immune surveillance, altering immune cell profiles, abnormal estrogen metabolism, and hormone signaling.^[11,13]^ Hence, research focused on the “microbiota-endometriosis-infertility” axis can deepen our understanding of EAI.

It is noteworthy that in animal models, transplantation of diseased gut microbiota can induce endometriosis.^[14]^ Moreover, the transplanted gut microbiota can further promote the growth and expression of inflammation in endometrial lesions.^[15]^ In contrast, models of endometriosis developed in mice and rhesus monkeys also manifest changes or dysbiosis in gut microbiota.^[16,17]^ Therefore, a simple causal relationship cannot be drawn between the 2. This paradox is also observed in clinical studies. For example, Moreno et al^[18]^ found that Atopobium (a common vaginal pathogen) is 1 of the culprits leading to infertile reproductive outcomes, whereas Ata et al^[19]^ found no Atopobium present in the vagina and cervix of individuals with endometriosis. This might be due to most previous studies being designed as case-control studies, where exposure time and outcomes are hard to ascertain. In observational studies, the association between gut microbiota and EAI can be influenced by confounding factors such as age, environment, dietary patterns, and lifestyle. Controlling these factors effectively in observational studies is challenging, limiting causal inference between gut microbiota and EAI.

Thus, using Mendelian randomization (MR) offers a novel approach to explore the causal relationship between gut microbiota and EAI.^[20]^ MR employs genetic variations to construct instrumental variables for exposures, determining causal relationships between exposures and disease outcomes.^[21]^ As different genetic sequences originate from genetic allocations, associations between genetic variations and outcomes are unaffected by common confounders. This makes it particularly apt for determining genuine causal relationships. MR has been widely used to probe the causal relationship between gut microbiota and diseases, including gynecological disorders,^[22]^ autoimmune diseases,^[20,23]^ and metabolic diseases.^[24]^ In this study, using summary statistics from genome-wide association studies from the MiBioGen and FinnGen consortia, a 1-way MR analysis was conducted to assess the causal relationship between gut microbiota and EAI.

## 2. Materials and methods

### 2.1. Data sources

Genetic variability data of the gut microbiome was sourced from the largest comprehensive genomic study of gut microbial composition published by the MiBioGen consortium.^[25]^ This study encompassed 16S rRNA gene sequencing spectra and genotypic data from 18,340 individuals from 24 countries including the USA, UK, Finland, Sweden, Denmark, Netherlands, etc. The summary data from this study covered 9 phyla, 16 classes, 20 orders, 35 families, and 131 genera of bacteria.

Summary statistics for endometriosis complicated with infertility were obtained from the FinnGen consortium.^[26]^ The phenotype “co-occurrence of endometriosis and infertility” was employed in the present study. This genome-wide association studies included 72,244 European adult subjects, with 1593 cases and 70,651 controls.

### 2.2. Instrumental variable selection

Gut microbiome composition was set as the exposure, and endometriosis complicated with infertility as the outcome. In addition, the analysis was carried out under 3 assumptions: The relevance assumption: Instrument variables (IVs) were associated with the exposure factors; The independence assumption: IVs were not related to any confounding factors; and The exclusion-restriction assumption: IVs only affected the outcome through the pathway of the exposure factors.^[27]^We adopted the criteria of *P* < 1 × 10^−5^ as the significance threshold based on recommendations from a study by Sanna et al,^[28]^ as the conventional significance standard (*P* < 5 × 10^−8^) is overly stringent. Genetic variations with close genomic locations tend to be inherited together, a phenomenon known as linkage disequilibrium. In this study, the threshold for linkage disequilibrium was set at *r*^2^ < 0.001 with a clumping window size of 10,000 kb. Palindromic single nucleotide polymorphisms (SNPs) were excluded. Additionally, the *P*-value of the instrumental variable in the outcome should satisfy *P* > 1 × 10^−5^, to avoid a direct association between the instrumental variable and the outcome.

### 2.3. Statistical analysis

Inverse variance weighted method was used as the primary analysis for MR. Additionally, 3 other methods were employed as references: the weighted median estimator, MR-Egger regression, simple mode, and weighted mode. Sensitivity analyses included tests for heterogeneity and pleiotropy. The significance of any pleiotropy was validated by the MR-Egger intercept test.^[29]^ Cochran Q test was employed for heterogeneity assessment, and *P* > .05 indicated no heterogeneity.^[30]^ Furthermore, we applied the “leave one out of analysis” method to detect any significant association dominated by a single SNP. Effect estimates for binary outcomes were represented as odds ratios with 95% confidence intervals. For the primary MR results, we applied the false discovery rate multiple testing correction at each taxonomic level (phylum, class, order, family, and genus).

**Figure 1.**
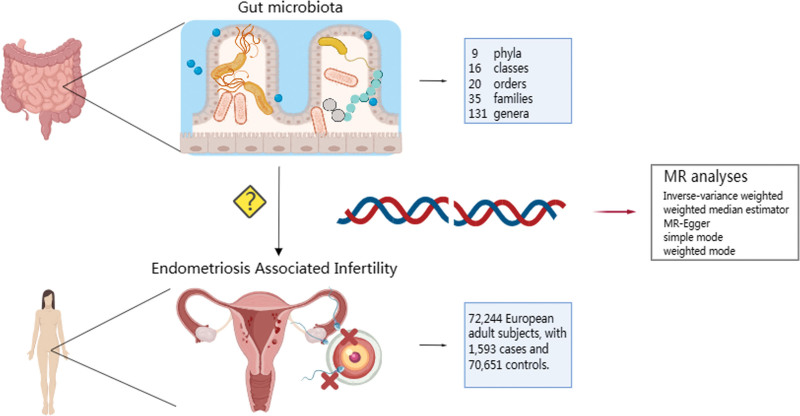
Schematic representation of the Mendelian randomization analysis exploring the association between gut microbiota and endometriosis associated infertility (EAI). Gut microbiota, highlighting the diversity of taxa analyzed, including 9 phyla, 16 classes, 20 orders, 35 families, and 131 genera. The central part of the figure illustrates the hypothesized influence of gut microbiota on EAI, represented by the silhouette of a female figure with highlighted reproductive organs indicating infertility. On the right, a DNA double helix symbolizes the genetic component of the study, with MR analyses methods listed, including Inverse variance weighted, Weighted median estimator, and MR-Egger. The bottom part of the figure provides details on the study population, comprising 72,244 European adult subjects, including 1593 cases of EAI and 70,651 controls.

We estimated the strength of the IVs using the *F*-statistic; where F < 10, weak instrumental variable bias was assumed. The *F* statistic (calculated as *F* = ([*n* − k − 1]/*k*) × (*R*^2^/[1 − *R*^2^]); *R*^2^ = 2 × EAF × (1 − EAF) × beta^2^, and indicating the proportion of variance in the exposure explained by the genetic variants; EAF = effect allele frequency; *n* = sample size; and *k* = number of IVs) indicates the strength of the relationship between the IVs and exposure.^[31,32]^ After removing the aforementioned noncompliant instrumental variables, we repeated the MR analysis to obtain the final MR estimate. Without heterogeneity and pleiotropy, inverse variance weighting (IVW) is a more reliable fitting model.^[33]^ For binary outcomes, the effect estimates are represented as odds ratios with a 95% confidence interval. The flowchart of the study is presented in Figure 1.

MR analysis was conducted in the *R* computing environment (version 4.1.2) using the TwoSampleMR package (version 0.5.6). *P* < .05 was considered statistically significant evidence of a causal effect.

## 3. Results

### 3.1. Instrumental variable selection and preliminary MR analysis

**Figure 2.**
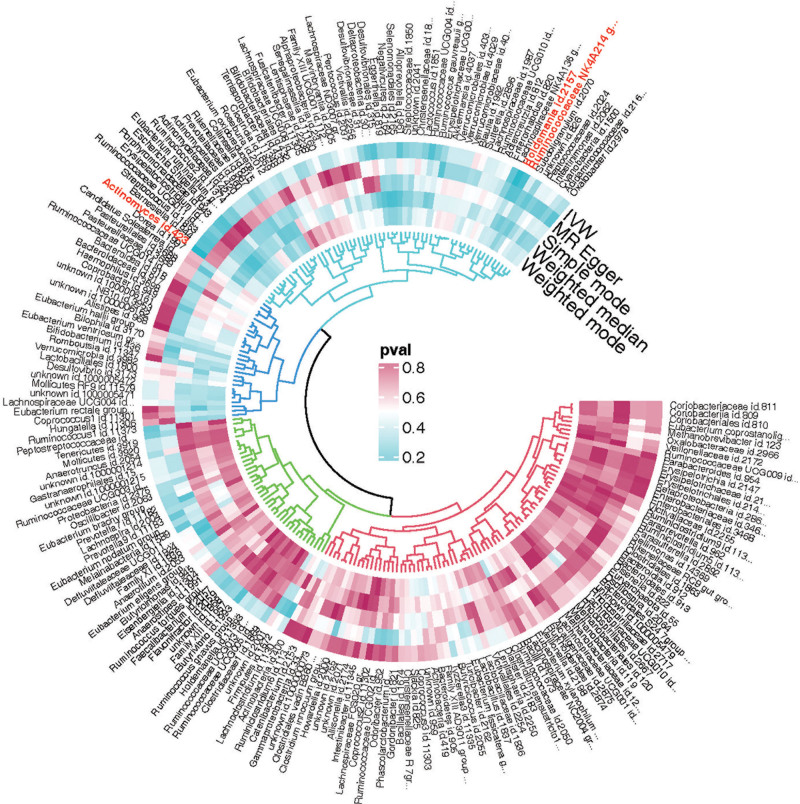
Phylogenetic tree and Mendelian randomization (MR) analysis of gut microbiota associated with EAI. Phylogenetic tree representing the association of various gut microbiota taxa with EAI as determined by MR analysis. The tree branches are labeled with the names of the microbiota taxa. The innermost ring indicates the taxonomic classification with different colors, while the surrounding rings represent *P*-value significance levels from multiple MR methods including inverse variance weighting (IVW), MR-Egger, and the Weighted Median approach. The color gradient from blue to red signifies increasing levels of significance, with red highlighting the taxa most strongly associated with EAI.

**Figure 3.**
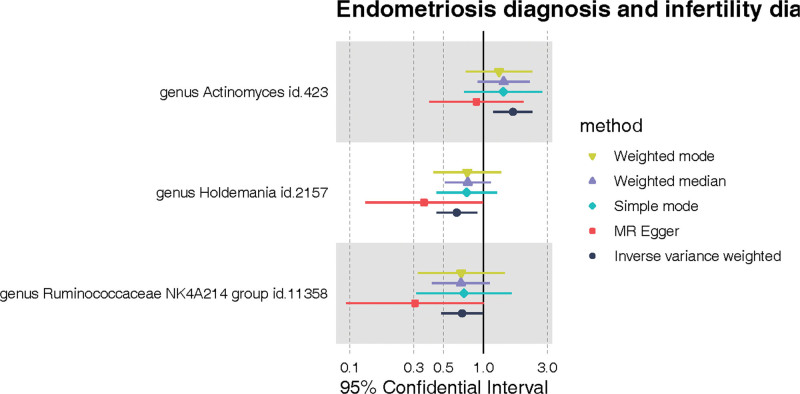
A forest diagram showing the gut microbiota associated with EAI. Forest plot demonstrates the strength and direction of associations between 3 genera of gut microbiota and the risk of endometriosis associated infertility (EAI), using different Mendelian randomization (MR) methods. Each horizontal line represents the 95% confidence interval (CI) for the odds ratio (OR) of a genus being associated with EAI. The genera *Actinomyces, Holdemania*, and the *Ruminococcaceae* NK4A214 group are shown with their respective ID numbers for reference. The plot symbols correspond to various MR methods: weighted mode (triangle), weighted median (diamond), simple mode (octagon), MR-Egger (circle), and inverse variance weighted (hexagon). A symbol to the right of the vertical line (OR > 1) suggests a risk factor, whereas a symbol to the left (OR < 1) indicates a protective factor. The analysis reveals that the genus *Actinomyces* is associated with an increased risk of EAI, while genera *Holdemania* and *Ruminococcaceae* NK4A214 group are associated with a decreased risk, signifying their potential protective effect.

Based on the criteria for selecting instrumental variables, a total of 2426 SNPs were chosen as tools for 211 types of gut microbiota. All selected SNPs had an *F* statistic >10, indicating no weak instrument bias. Figure 2 shows the relationship between 211 bacterial taxa and EAI; Based on the IVW analysis, 3 bacterial taxa showed statistical significance in Figure 3. Among them, the genus Holdemania and genus Ruminococcaceae NK4A214 group demonstrated protective effects, while the effects of genus *Actinomyces* was opposite.

### 3.2. Detailed MR analysis

As depicted in Figure 3, IVW suggested that genetic prediction of the genus *Actinomyces* (OR = 1.657, 95%CI: 1.187–2.312, *P* = .00298) was associated with an elevated risk of EAI. In contrast, genus Holdemania (OR = 0.630, 95%CI: 0.444–0.894, *P* = .00969) and genus Ruminococcaceae NK4A214 group (OR = 0.689, 95%CI: 0.481–0.999, *P* = .0439) were found to be protective factors against EAI.

### 3.3. Sensitivity analysis

Results of the sensitivity analysis are presented in Tables 1 and 2. Cochran Q test found that test values for 6 bacterial taxa were >0.05, indicating no heterogeneity among the instrumental variables. Additionally, the intercept from the MR-Egger test showed no significant difference from zero, confirming the absence of horizontal pleiotropy. Meanwhile, the leave-one-out results further validated data robustness (Supplementary Figure 1, http://links.lww.com/MD/L839), suggested that no single SNP drastically influenced the overall results, reinforcing the robustness of these MR outcomes.

**Table 1:** Heterogeneity score for Mendelian randomization to analyze the association between gut microbiota and endometriosis associated infertility.

Exposure	Q_test			
	Method	Q	Q_df	Q_*P* value
genus.*Actinomyces*.id.423	MR-Egger	0.972	5	0.965
	IVW	3.733	6	0.713
genus.*Holdemania*.id.2157	MR-Egger	18.9	12	0.090
	IVW	21.1	13	0.072
genus.*Ruminococcaceae*NK4A214group.id.11358	MR-Egger	7.23	11	0.780
	IVW	9.19	12	0.686

IVW = inverse variance weighted, MR = Mendelian randomization

**Table 2:** Pleiotropic score for Mendelian randomization to analyze the association between gut microbiota and endometriosis associated infertility.

Exposure	MR-Egger intercept test		
	Egger_intercept	Se	*P* value
genus.*Actinomyces*.id.423	0.078	0.047	0.157
genus.*Holdemania*.id.2157	0.058	0.05	0.269
genus.*Ruminococcaceae*NK4A214group.id.11358	0.062	0.044	0.189

### 3.4. Additional results

No heterogeneity or horizontal pleiotropy was observed among other bacterial taxa. Scatter plots illustrate the effect trends across various MR methods, and no significant outliers were observed (Supplementary Figure 2, http://links.lww.com/MD/L840). Funnel plots for each bacterial taxa demonstrated symmetrical distribution around the effect sizes, further validating the unbiased nature of the IVW model (Supplementary Figure 3, http://links.lww.com/MD/L841).

## 4. Discussion

We applied MR to analyze 211 common microbial groups in the gut. Our results revealed that genus *Actinomyces* (OR = 1.657, 95% CI: 1.187–2.312, *P* = .00298) was associated with an elevated risk of EAI. In contrast, genus *Holdemania* (OR = 0.630, 95%CI: 0.444–0.894, *P* = .00969) and genus *Ruminococcaceae* NK4A214 group (OR = 0.689, 95% CI: 0.481–0.999, *P* = .0439) were found to be protective factors against EAI.

Previous studies have posited significant differences in the gut microbiota between EAI patients and healthy women.^[19,34]^ Moreover, interventions with antibiotics or probiotics have shown potential in ameliorating EAI,^[15,35,36]^ highlighting the close relationship between gut microbiota alterations and EAI. However, given the inherent limitations of observational studies or animal experiments, these investigations could not definitively pinpoint the exact roles of various gut microbial groups in EAI.^[37]^ Our comprehensive analysis of 211 microbial taxa offers clearer insights into the potential beneficial or detrimental impacts of specific bacteria on EAI patients, deepening our understanding of EAI pathogenesis and paving the way for novel therapeutic strategies. Notably, common bacteria in EAI research, such as lactobacillus, Firmicutes, and Proteobacteria, were not found to have a causal relationship with EAI. This calls for a more cautious interpretation of past studies, suggesting possible clinical factors might have influenced our observational conclusions.

Interestingly, in our findings, the genus *Holdemania* was introduced for the first time as having a protective role against EAI. Prior to our investigation, no research had reported variations of the *Holdemania* bacteria in EAI or endometriosis patients. *Holdemania* is an anaerobic Gram-positive bacteria similar to Clostridium. Previous research has indicated that *Holdemania* abundance correlates positively with dietary fiber intake and overall polyunsaturated fatty acid consumption, including both ω-3 and ω-6 polyunsaturated fatty acid, classifying it as a beneficial bacteria.^[38]^ Concurrently, *Holdemania* is closely related to the increased production of short-chain fatty acids (SCFAs), which mainly include acetate, propionate, and butyrate. These SCFAs play vital roles in maintaining gut homeostasis, immunity, and anti-inflammatory actions, thus aiding in the amelioration of inflammation-related chronic diseases.^[39,40]^ Given that EAI fundamentally is a chronic systemic inflammatory syndrome, we hypothesize that *Holdemania* might exert its anti-inflammatory protective role in EAI through the augmentation of SCFAs in the gut. The genus *Ruminococcaceae* NK4A214 group has been previously linked to endometriosis, with a decline in its abundance observed in patients. Enhancing its prevalence has been proposed to assist in treating endometriosis.^[41]^ Some studies have indicated that *Ruminococcaceae* inversely correlates with apoptosis of murine intestinal epithelial cells and IL-6 levels.^[42]^ Thus, an increased abundance of *Ruminococcaceae* may help reduce pelvic inflammation, subsequently improving the manifestations of EAI. Our research further corroborates this conclusion from a causal perspective.

On the other hand, in our research, we identified that gut microbiota from the genus *Actinomyces* might induce or exacerbate EAI. Additionally, it has been established that there is a notable change in the abundance of *Actinomyces* in patients with Endometriosis. Hence, it’s not surprising that it could play a role in the pathogenesis of EAI.^[43]^ However, what is intriguing is a previous study found that the abundance of *Actinomyces* in the peritoneal fluid of Endometriosis patients tends to decrease.^[43]^ This contrasts with our findings, a discrepancy that may be attributed to differences in microbial tissue sources. Our results suggest that the genus *Actinomyces* could potentially trigger or exacerbate EAI. *Actinomyces* is nonmotile, filamentous, Gram-positive, and obligately anaerobic. It is also an opportunistic pathogen and commensal of the oral cavity, pharynx, gut, urogenital tract, and skin.^[44]^ Previous studies have shown that while *Actinomyces* typically isn’t pathogenic in humans, the alterations it causes in the gut environment and immune factors might aggravate inflammation-induced damage.^[45,46]^ Our results propose that it might worsen EAI. This conclusion is based on causality, and further expansive studies are required to clarify its mechanism. It’s essential to note that our study focused on entire microbial taxa at the genus level. Different species within the same genus might possess varied pathological or physiological functions, cautioning us against potential heterogeneity.

In essence, the potentially complex interactions among gut microbial communities might explain discrepancies between genetic predictions and clinical observations. We speculate that a significant reason might be the involvement of gut microbiota in inflammation and immune regulation, thus playing a part in the entire pathophysiological process of EAI. However, our conclusions need verification through further prospective randomized controlled trials. Specifically, research should be undertaken on the potential of gut probiotic interventions or antibiotic treatments for EAI. We are the first to conduct a large-scale MR analysis on the causal relationship between gut microbial communities and EAI, but certain limitations are still inevitable. We acknowledge that even though our study analyzed common gut microbial communities, the gut microbiome is vast and highly heterogeneous. Furthermore, our research only scrutinized populations from Europe, which means caution is required when generalizing our findings to other demographics. Additionally, even though we’ve analyzed the genetic causal relationships between the gut microbiome and EAI independently, the intricate interplay between EAI and the gynecological, reproductive, and endocrine systems with the gut microbial communities still warrants consideration.

## 5. Conclusions

Conclusively, our study underscores the potential protective effects of the genus *Holdemania* and the genus *Ruminococcaceae* NK4A214 group in mitigating EAI. Conversely, gut microorganisms from the genus *Actinomyces* could potentially trigger or amplify EAI. While the genus *Actinomyces* is frequently recognized as beneficial, individual species and strains within this genus might elicit varying influences on human health. As such, a deeper exploration into the roles and underlying mechanisms of these bacterial genera in the progression of EAI is imperative.

## Author contributions

**Funding acquisition:** Wangshu Li.

**Validation:** Yan Wang.

**Writing—original draft:** Yan Wang, Wangshu Li, Chunfang Ha.

**Writing—review and editing:** Yan Wang, Wangshu Li.

## Supplementary Material






