# Supplementary figures and images for: A large-scale causal analysis of gut microbiota and endometriosis associated infertility: A Mendelian randomization study
Source: Medicine (Baltimore). 2024 Mar 22;103(12):e37383. doi: 10.1097/MD.0000000000037383 (PMC10956985; doi:10.1097/MD.0000000000037383)

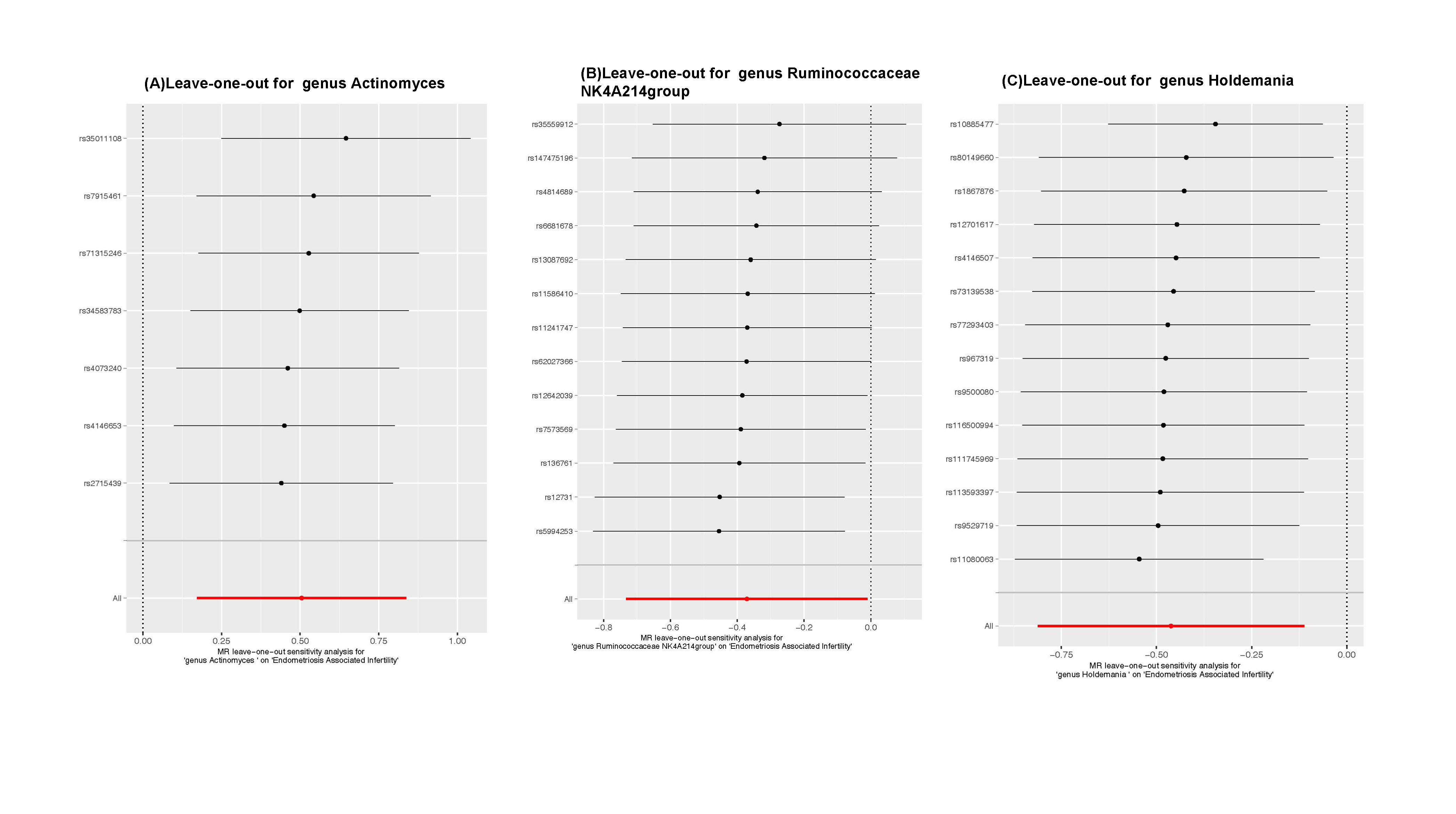

Supplement: Supplementary file 1 [file medi-103-e37383-s001.tif]

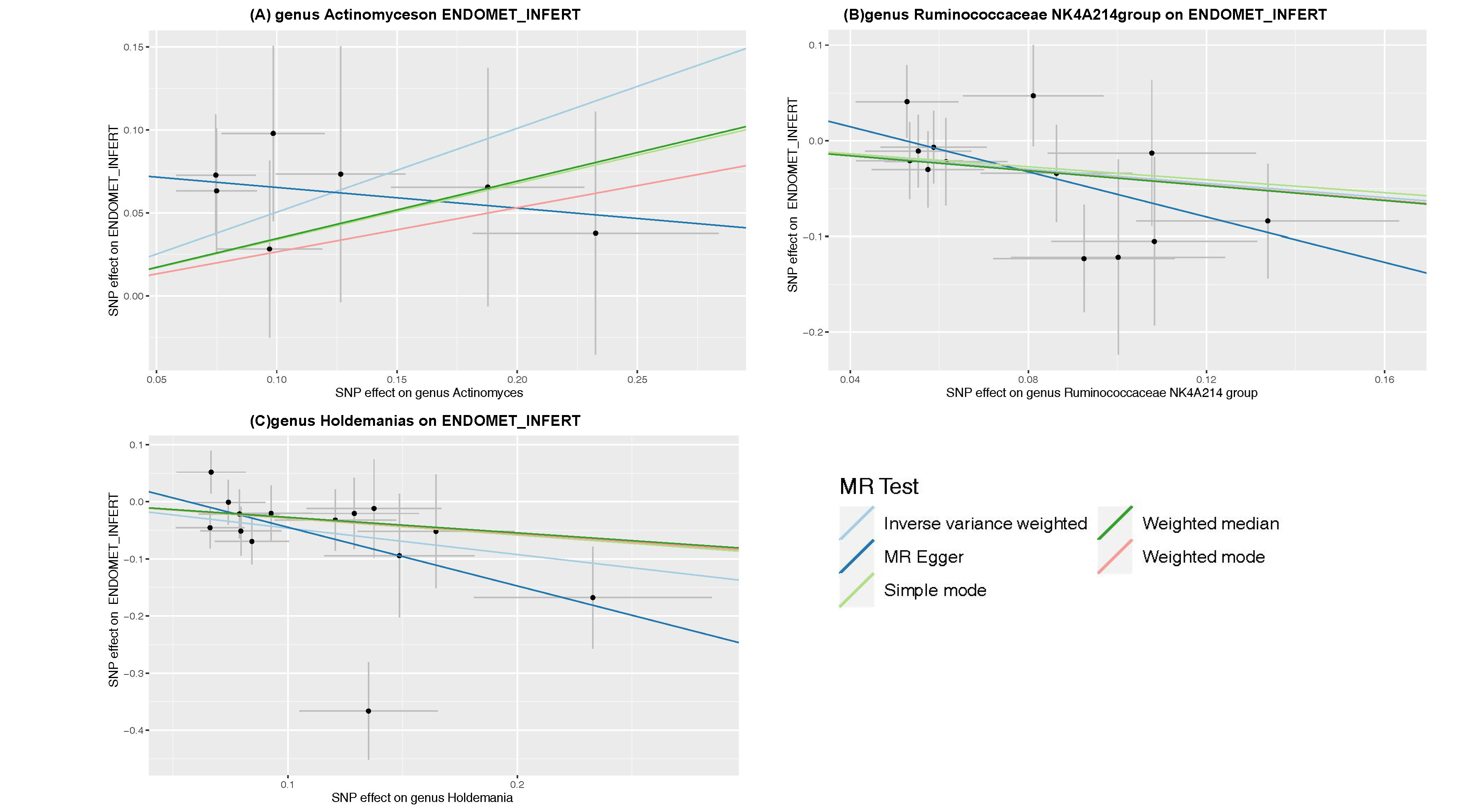

Supplement: Supplementary file 2 [file medi-103-e37383-s002.tif]

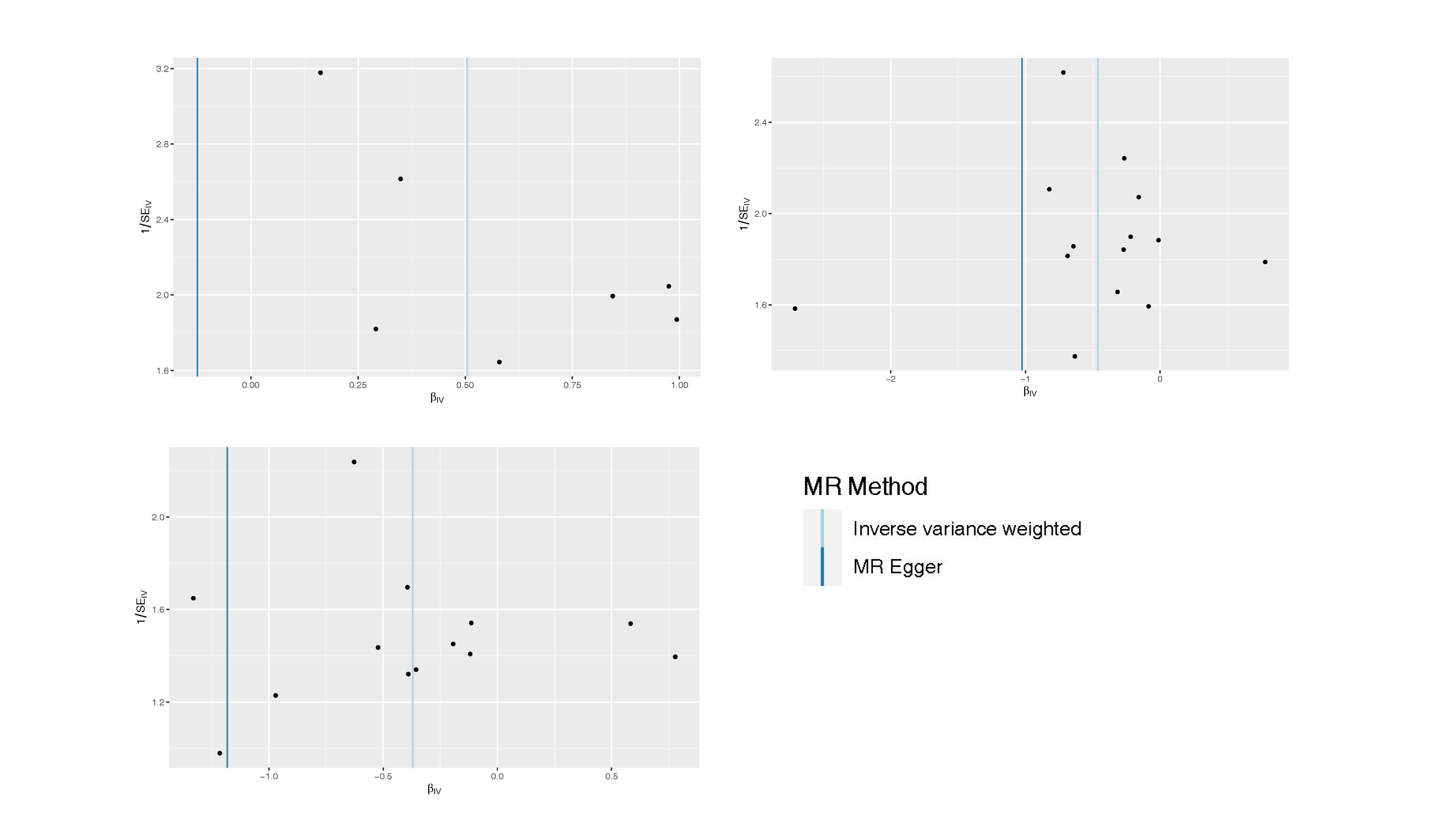

Supplement: Supplementary file 3 [file medi-103-e37383-s003.tif]
